# Tensile and Flexural Properties of Cement Composites Reinforced with Flax Nonwoven Fabrics

**DOI:** 10.3390/ma10020215

**Published:** 2017-02-22

**Authors:** Josep Claramunt, Heura Ventura, Lucía J Fernández-Carrasco, Mònica Ardanuy

**Affiliations:** 1 Departament d’Enginyeria Agroalimentària i Biotecnologia, Universitat Politècnica de Catalunya, Barcelona 08034, Spain; josep.claramunt@upc.edu; 2 Departament de Ciència dels Materials i Enginyeria Metal·lúrgica, Universitat Politècnica de Catalunya, Barcelona 08034, Spain; heura.ventura@upc.edu; 3 Departament d’Enginyeria Civil i Ambiental, Universitat Politècnica de Catalunya, Barcelona 08034, Spain; lucia.fernandez@upc.edu

**Keywords:** cement composites, nonwoven fabrics, tensile properties, mechanical behavior

## Abstract

The aim of this study is to develop a process to produce high-performance cement-based composites reinforced with flax nonwoven fabrics, analyzing the influence of the fabric structure—thickness and entanglement—on mechanical behavior under flexural and tensile loadings. For this purpose, composite with flax nonwoven fabrics with different thicknesses were first prepared and their cement infiltration was evaluated with backscattered electron (BSE) images. The nonwoven fabrics with the optimized thickness were then subjected to a water treatment to improve their stability to humid environments and the fiber-matrix adhesion. For a fixed thickness, the effect of the nonwoven entanglement on the mechanical behavior was evaluated under flexural and direct tension tests. The obtained results indicate that the flax nonwoven fabric reinforcement leads to cement composites with substantial enhancement of ductility.

## 1. Introduction

Natural fibers have been extensively studied as reinforcement for cementitious materials since they provide adequate stiffness, strength, and bonding capacity to enhance their flexural strength, toughness, and crack resistance [1]. In the majority of the studies about cement composites reinforced with natural fibers, the reinforcement is in the form of short fibers (usually pulps) randomly dispersed on the matrix [2,3,4,5,6,7,8,9,10] and only a few studies describe the performance of composites with the fibers in the form of textile structures, like directional, woven, or nonwoven fabrics [11,12,13,14,15]. The randomly dispersed fibers allow significant improvements on the flexural strength and ductility of the cement composites, but these improvements are limited by the short length of the fibers (usually in the order of 2–10 mm to few cm) and the maximum quantity that it is possible to mix with the cement matrix [1]. In the composites reinforced with textile structures, apart from the improvements on the flexural strength, there is also a significant enhancement of the tensile strength and ductility under tensile deformation [13]. In this class of composites the reinforcement is in the form of a textile structure, and its reinforcement capacity does not only depend on the fiber intrinsic mechanical properties, fiber content, fiber orientation, fiber form (staple or continuous filament), yarn structural form (if the fiber is combined with other fibers forming a yarn), but also on the fabric structure (e.g., how the fibers of yarns are interlaced) [16]. The most common fabric structures used for the reinforcement of cement composites are very open directional fabrics (with the filaments aligned in one or more direction) or woven fabrics (with the fibers in the form of yarns distributed usually in warp and weft directions) [17,18,19,20,21,22,23,24]. Nonwoven fabrics, where the fibers are randomly distributed in the form of mats, are much less often applied in the cement composites since they are more closed structures with higher difficulty for infiltration of the cement matrix through the reinforcement. On the nonwoven fabrics, the interlacing is done by a chemical, thermal, or mechanical entanglement of the fibers. The mechanical bonding processes, as needle-punching, allows the fabrication of structures with very high mechanical strength at very low cost. Moreover, in these fabrics, the fibers can be aligned in X, Y, and Z directions. In these fabrics, the mechanical properties are mainly determined by the fibers used, their orientation, and also by the degree of fiber entanglement. In this sense, the development of high-strength nonwovens (it is to say, with high degree of fiber entanglement) from natural fibers is the first step to produce natural-fiber reinforced cement composites for low thickness panels for structural applications.

In this sense, although nonwoven fabrics made of natural fibers have been extensively applied in composite reinforcement for polymeric matrices [25,26,27,28], their application in a cementitious matrix has been scarcely reported and even less using natural fibers to prepare the nonwoven fabrics. Apart from our own previous work [12], as far as we know, there are not results available in the literature about the characterization of the tensile and flexural properties of Portland cement composites reinforced with nonwoven fabrics made of natural fibers like flax. The applications of these materials could be the same as conventional fibrocement (roof plates, dry partition walls, plates for ventilated façades, urban furniture, among others) but with higher performance, enabling separation between the anchor points or support for higher loads. 

In this paper, a process to produce high-performance cement-based composites reinforced with nonwoven flax fabrics is presented, analyzing the influence of the nonwoven structure—higher or lower entanglement—on the mechanical performance and cracking under tensile and flexural loadings.

## 2. Results and Discussion

### 2.1. Effect of Nonwoven Thickness on the Cement Penetrability of the Nonwoven Fabrics

**Figure 1:**
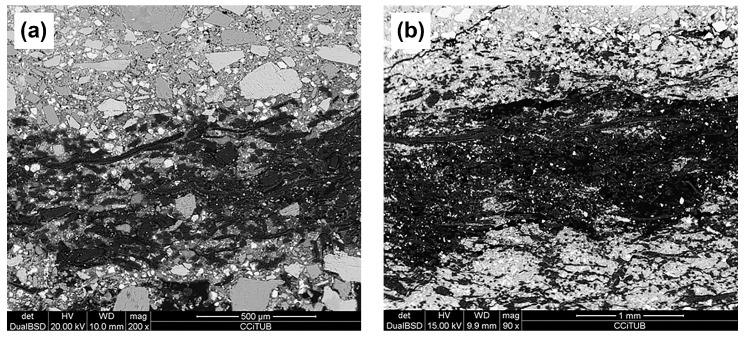

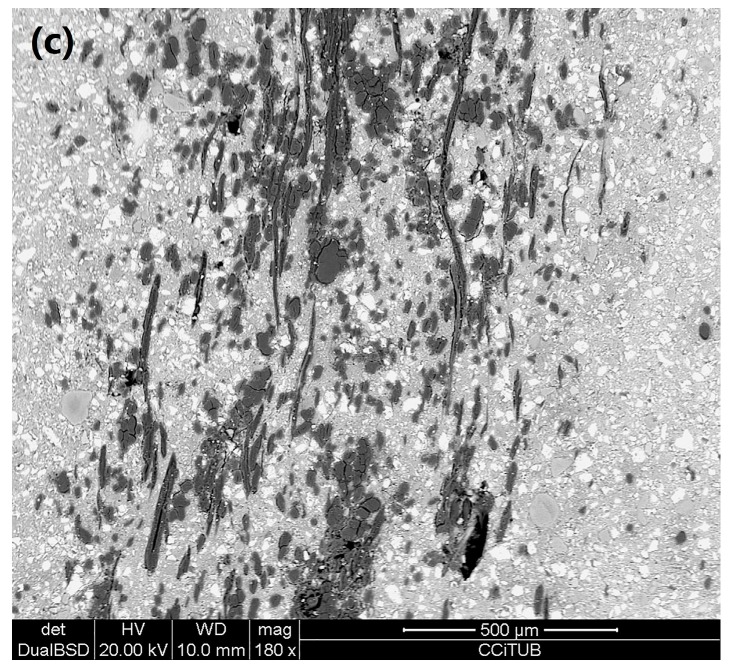
BSE–SEM images of the cement composites: (**a**) with sand; (**b**) with a nonwoven of 9 mm thickness and 680 g/m^2^; (**c**) with a 2 mm thickness and 275 g/m^2^ nonwoven.

During the first attempts to prepare the composites, some experimental difficulties appeared. The first one was due to the heterogeneity of the morphology of the components of the cement matrix (cement and sand). The sand with higher particle size distribution tended to accumulate on the nonwoven surface, as shown in Figure 1a. The solution proposed was to eliminate the sand in the matrix composition. On the other hand, the effect of the thickness and areal weight of the nonwoven fabrics was analyzed in order to know which ones led to the greater uniformity and compactness of the resulting compounds. For this purpose, as is detailed on the Section 3.1, four types of nonwovens with the same entanglement with different thickness and areal weight were prepared. As can be expected, it was found that the structures with higher weight and thickness did not allow good infiltration of the cement particles into the porous structure of the nonwoven fabrics (Figure 1b). However, using the nonwoven fabric with the lowest areal weight and thickness (2 mm thick and 275 g/m^2^), it was possible to prepare more homogeneous composites, with a good matrix infiltration through the fabric, as shown in Figure 1c.

Taking into account the BSE-SEM images, the sample with the lowest weight and thickness was used to prepare the cement composites (sample of 2 mm thickness and 275 g/m^2^). 

### 2.2. Effect of Number of Layers and Water Treatment of the Nonwovens on the Composite Performance

To analyze the effect of number of layers and water treatment of the nonwovens on the composite performance, 1 cm thick cement composite specimens reinforced with three or four layers of fabrics and in the case of four layers with water treatment or untreated nonwovens were prepared. As previously mentioned, the matrix used was neat Portland cement without sand, being fixed an initial water:cement (w/c) ratio of 2:1. The references and the composition of the prepared composites are presented in Table 1.

**Figure 2:**
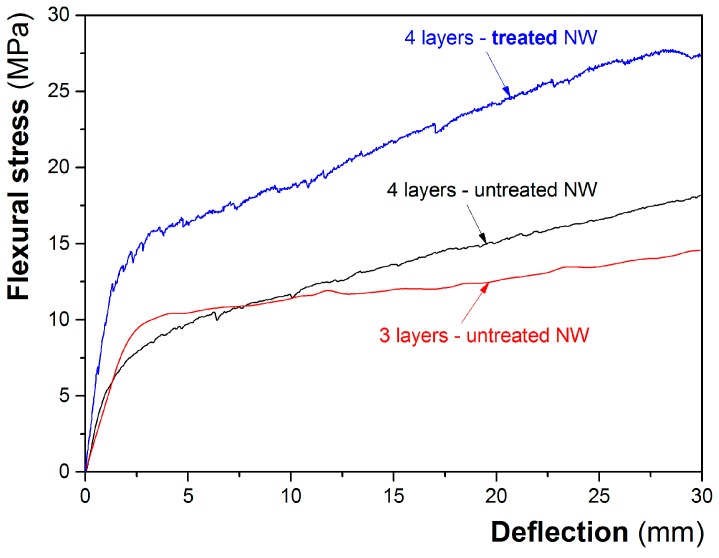
Typical flexural strength versus deflection curves of the cement composites reinforced with three and four layers of untreated nonwovens, and the composite reinforced with four layers of water-treated nonwoven fabrics.

Figure 2 shows the typical load-deflection curves for the specimens analyzed under flexural tests.

As can be seen, all the composites showed very post failure behavior under flexural configuration, with a high capacity for deformation, with the formation of multiple cracking. As reported in Table 1, with the increased number of layers, there is a slight increase in the flexural strength and fracture energy. Moreover, an increase on the slope on the part of the curve corresponding to the effect of the reinforcement can be observed, which increases with the number of layers, indicating a higher stiffness of the material during the stress state. On the other hand, for the same number of layers, the composites prepared with the treated nonwoven fabrics presented a considerably higher flexural strength and fracture energy than the corresponding ones, prepared with the untreated fabrics. In agreement with previous results obtained by our research group [12], these results demonstrate the beneficial effects of the water treatment of the nonwovens on the mechanical behavior of the composites. Moreover, this treatment will prevent the loss of fiber-matrix bonding, induced by dimensional changes in the vegetable fibers.

### 2.3. Effect of Needle-Punching of the Flax-Fiber Nonwovens in the Composite Properties

Once fixed, the composition of the cement composites (with four layers and with the water treated nonwoven fabrics mixed with Portland cement with a nonwoven:cement ratio of around 9 wt %; the effect of the degree of interlacing or entanglement of the nonwoven fabrics on the final properties of the composites was analyzed. For this purpose, the nonwoven fabrics prepared were subjected to high (named HNP-NW) or medium (named MNP-NW) entanglement of the fibers on a needle-punching machine (see details of the preparation on Section 3.2).

Firstly, the properties of the two nonwoven fabrics prepared with the two entanglement degrees are presented in Table 2.

As can be seen, and as it is expected, increasing the needle-punching intensity increases the average maximum force at break of the fabrics from about 18 N to about 40 N (more than 200%) meanwhile the deformation at break decreases only from 48% to about 39% (about 23%). This is related with the higher entanglement between the fibers due to the effect of higher needle stroke speed and, on the other hand, with the higher densification of the material (with a weight increase of about 25% combined with a thickness decrease of about 30%). 

**Figure 3:**
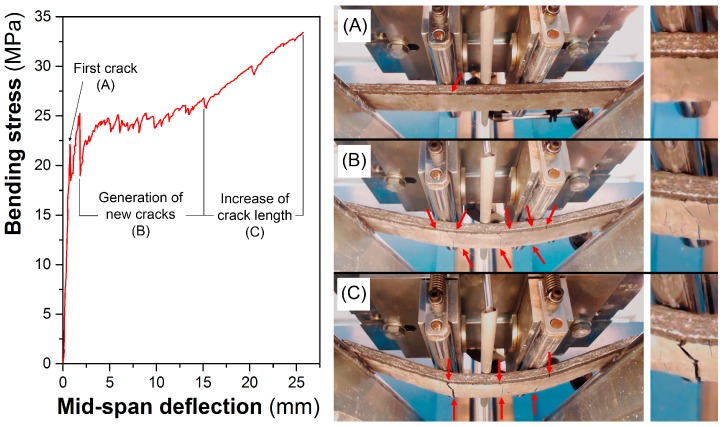
Representative curve and cracking phases of the composites obtained under flexural stress: (**A**) first crack; (**B**) generation of new cracks; (**C**) increase of crack length.

Concerning to the flexural behavior of the cement composites prepared with the HNP-NW and MNP-NW nonwoven fabrics, Figure 3 shows the typical bending stress vs. mid span deflection curves and the cracking phases obtained under flexural configuration. As shown on the curves, the initial behavior in bending was linear and with a high slope until the first crack appeared (zone A). In this zone, the composite works by maintaining compatibility of deformations, i.e., the reinforcement and the matrix deform exactly the same length, so that the resulting stress depends on the amount of material and its modulus of elasticity. In this case, as the volume fraction of the fibers is 15% (lower than 30%) and the deformability of the nonwoven fabric is higher than >30% (see Table 2), the mechanical characteristics of the composite correspond more to those of the matrix than to the fiber [13,29,30].

Afterwards, the failure of the composites was characterized by a multiple cracking formation (zone B). In this zone, the material works without compatibility of deformations since the matrix absorbs most of the compression forces on the upper part of the specimen and the reinforcement absorbs most of the tensile forces on the nether part of the specimen. Under these conditions, the stress transfer mechanism is achieved by the reinforcement-matrix adhesion in the zones between cracks [30]. If there is a low fiber-matrix adhesion, once the first crack appears there is a deboning of the fibers and fracture of the composite. If the adhesion between the two phases is very high, the nether part can deform as long as reinforcement does not break, opening evenly distributed numerous cracks. For the composites under study, the appearance of many fine cracks demonstrates a good reinforcement-matrix bonding for both the composites prepared with the HNP-NW and MNP-NW nonwoven fabrics. Finally, once all of cracks have been produced, the behavior of the material is defined by a widening of the cracks characterized by a smoothing of the curve (zone C). 

**Figure 4:**
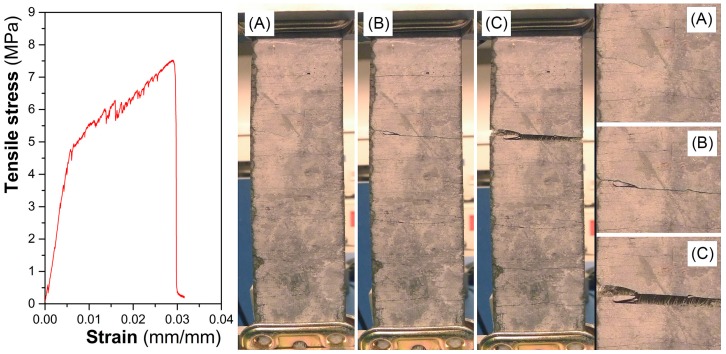
Representative curve and cracking phases of the composites obtained under tensile stress: (**A**) first crack; (**B**) generation of new cracks; (**C**) increase of crack length.

In a similar way, the cracking behavior can be observed in Figure 4 for tensile configuration.

**Figure 5:**
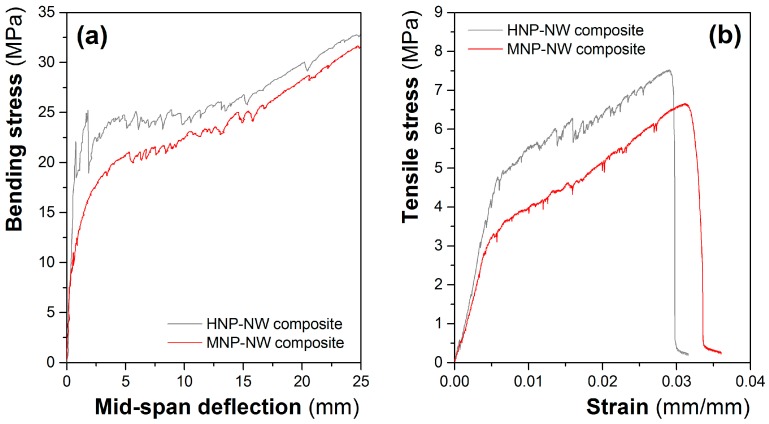
Typical bending (**a**) and tensile curves (**b**) obtained for the composites prepared with HNP-NW and MNP-NW nonwovens.

Concerning the results obtained for the two kinds of nonwoven fabrics, the typical bending and tensile curves for the composites are presented in Figure 5. Comparing bending and tensile responses it can be seen that the limit of proportionality (LOP) and modulus of rupture (MOR) occur at higher stress levels for bending configuration, while toughness and stiffness modulus (E_zone_ modulus) values were higher for tensile configuration.

The average values and standard deviation of LOP, MOR, E modulus, and toughness, determined and obtained from the flexural and tensile tests, are compiled in Table 3 and Table 4. 

Concerning the effect of the entanglement of the nonwoven used as reinforcement, in general, the HNP-NW led to a composite with higher values of LOP, MOR, E modulus, and toughness. For both flexural and tensile tests, the end of the linear elastic range, delimited by the LOP, was higher for the composite reinforced with HNP-NW. The composites prepared with HNP-NW presented average LOP values of 5.54 and 2.67 MPa for flexural and tensile tests, respectively, while for MNP-NW composites, the LOP values were 18.05 and 5.06, respectively. The stiffness modulus measured (E_zone_) for bending and tensile tests was 11.09 and 15.08 MPa, respectively, for HNP-NW, while for MNP-NW composites it was 15.71 and 18.17 MPa. Since in this linear range the response is dominated by the matrix [13], this increase in LOP and E_A_ modulus values for the composite prepared with the HNP-NW could be related to a higher content of cement on these composites. This is because the HNP-NWs are thinner than the MNP-NWs, and, for the same weight percentage of reinforcement, more matrix content is necessary to have the same thickness of the plates.

Focusing on the post-cracking behavior (zone B and zone C), the increase of the flexural strength from the stage of the first crack appearance (LOP value) until the failure (MOR value), was of 25.54 and 15.36 MPa for the composites prepared with the MNP-NW and HNP-NW respectively. This means that there is an increase of 461% and 86% of the flexural strength of the MNP-NW and HNP-NW composites respectively by the effect of the reinforcement. The significant increase observed for MNP-NW composite, found also in direct tensile tests but with lower intensity, can be related with a lower LOP value due to that, as previously mentioned, these composites have higher cement content for a same weight percentage of reinforcement. Nonetheless, on the zone C the differences on the increase of the strength are lower since they have the same reinforcement content and the matrix is saturated with cracks. 

On the other hand, the E_B_ values on the zone B were 0.306 GPa for the composites prepared with MNP-NW and 0.517 for the ones prepared with HNP-NW, meanwhile for the zone C were 0.331 and 0.338 GPa, respectively. This result indicates that the increases of E_zone_ values on the composites prepared with the MNP-NW mainly take place on the zone B, meanwhile on the zone C are similar for both composites.

For the tensile behavior, the differences between the two systems were very small, being the values of 0.144 and 0.156 GPa on the zone B and 0.141 and 0.152 GPa on the zone C for the composites prepared with MNP-NW and HNP-NW, respectively. These results, combined with the appearance of the curves, could be interpreted as that under flexural forces the first cracking process and later crack widening are defined by two zones. Meanwhile, under tensile forces, these two phenomena are produced simultaneously and, hence, on the tensile curve zones B and C are undifferentiated. 

As is shown in toughness values compiled in Table 3 and Table 4, the composites prepared with HNP-NWs presented higher average toughness of about 30% and 10%, for bending and tensile configurations, respectively, than the ones prepared with MNP-NWs. MOR values followed the same trend, with increases around 8% for NHP-NW composites under bending and tensile configurations. These results can be related to the higher entanglement of the HNP-NW nonwovens, which leads to higher strength and deformation of the composites.

**Figure 6:**
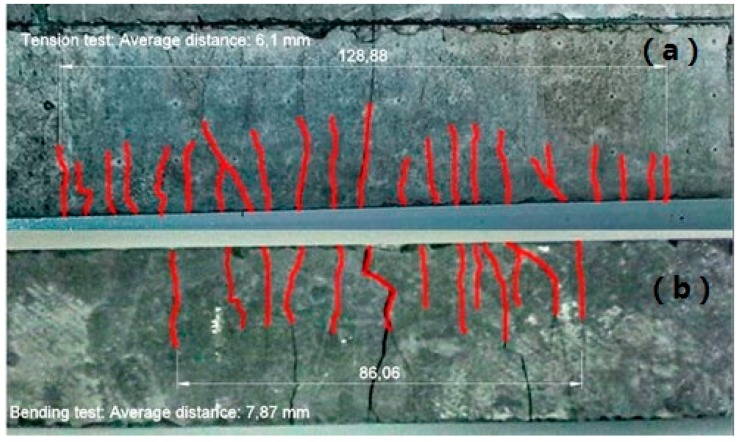
Cracking maps of two composites from the same plate tested under tensile (**a**) and bending test (**b**).

With respect to the cracking phases, comparing both mechanical configurations, the first crack appeared at lower values of strength and deformation under tensile than under flexural tests, and, as can be seen in Figure 6, the crack spacing was higher for the specimens tested under bending stress than the ones tested under tensile stress (7.9 and 6.1 mm, respectively). On the other hand, the cracking pattern was irregular, and partially followed the tensile direction for both the bending and tensile tests. This irregularity is related to the anisotropic distribution of the fibers on the nonwoven specimen.

## 3. Materials and Methods

### 3.1. Materials

UNE-EN 197-1:2011 Type I cement, supplied by CimenCat (Girona, Spain), was used as the matrix. Following previous research, 10 wt % of silica fume sourced from Sika replaced the cement, to ensure the total conversion of portlandite (Ca(OH)_2_) in hydrated calcium silicate [31]. Quartz flour sand was supplied by Sibelco Hispania (Barcelona, Spain). Sika Viscocrete-3425 fluidizer, obtained from Sika S.A.U. (Madrid, Spain), was used at a maximum dosage rate of 4 g/100 g of cement, to aid workability.

Flax-cottonized fibers (average length of 5.9 cm), provided by Institut Wlokien Naturalnych (Poznan, Poland), were used to prepare the nonwoven structures.

### 3.2. Nonwoven Preparation and Characterization

**Figure 7:**
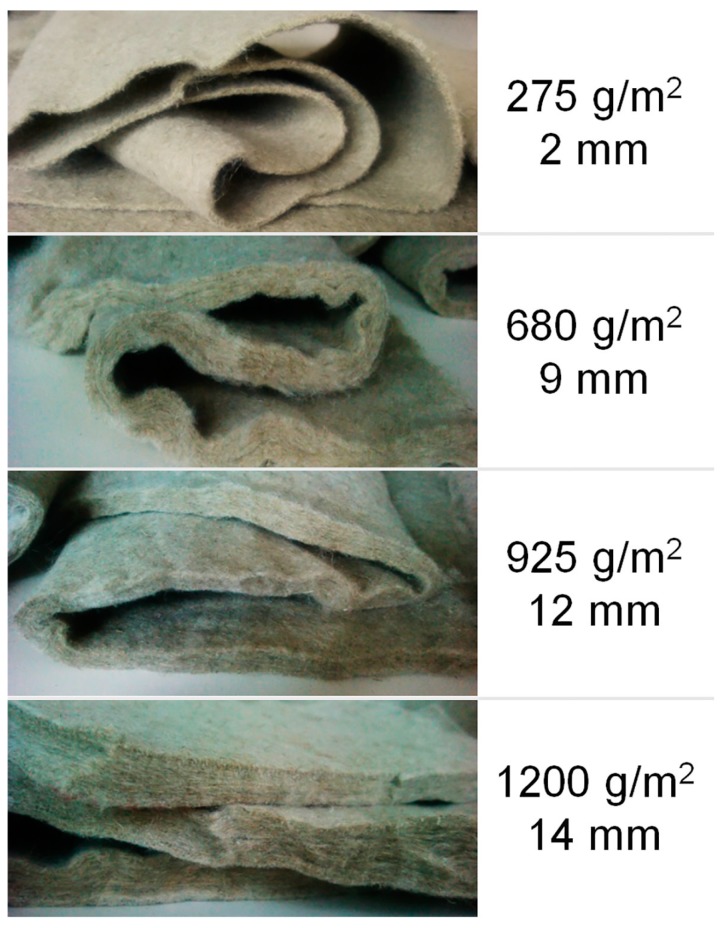
Photographic images of the nonwoven fabrics prepared.

Nonwoven fabric samples were prepared on a pilot plant, double needle-punching machine DILO OUG-II-6, equipped with universal card clothing, cross-lapper, batt feeder, and needle-punching loom. The flax fibers were first opened and carded to form a thin web, which was laid by the cross-laying method to form batts. These batts were consolidated on the needle-punching to form the nonwoven mats. In order to determine the optimized nonwoven structure, which could allow a good penetrability of the cement on the porous structure of the nonwoven fabrics, four types of nonwovens with the same entanglement—but with different thickness and areal weight—were prepared (Figure 7). Once the optimized nonwoven structure was selected, two types of nonwovens with different entanglement were prepared (named as HNP-NW, high needle-punching nonwoven; and MNP-NW, medium needle-punching nonwoven). The HNP-NW and MNP-NW were produced with a needle stroke of 750 and 600 r.p.m. respectively. The machine parameters to prepare these nonwovens were determined in previous research [32]. 

Based on previous research [8], a water treatment was performed on the nonwovens. This treatment consists on: (1) wetting by soaking overnight in water bath at environmental temperature; and (2) drying in oven with air recirculation at 60 °C for 4 h. These steps were repeated four times.

Tensile tests (based on UNE-EN ISO 13934-1 Standard) were performed to determine the changes in the breaking force of the nonwovens. The tests were done in a MTS machine with a load cell of 5 KN, using a displacement rate of 100 mm/min. The nonwovens were tested in both a machine direction (MD), and a cross-direction (CD). 

All the nonwoven fabrics were conditioned at a standard temperature and relative humidity of 20 ± 2 °C and 65% ± 2%, respectively, prior to testing. 

### 3.3. Composite Preparation and Characterization

**Figure 8:**
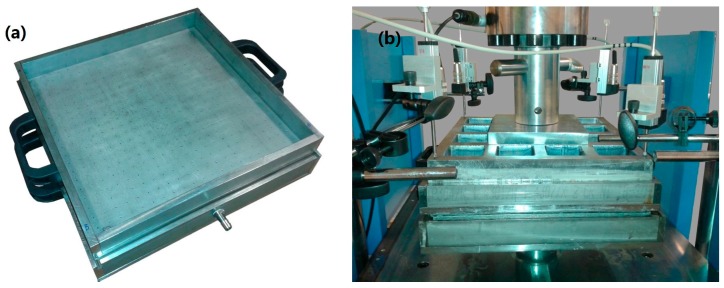
Photographic images of the mold used to prepare the composites (**a**) and the pressing process used for its compaction (**b**).

Laminates with nonwoven cross-oriented layers (with the nonwoven fabrics in machine direction or cross direction) were produced as is described following: first, a filter fabric is placed on the bottom of the mold to retain the cement paste particles. Then, the mold is filled with the cement paste to a height of about 3 mm and filtered with the vacuum pump to remove the excess of water. After this, a nonwoven layer impregnated with cement paste is placed in the mold and pressed with a roller at the same time that the excess of water is removed with the vacuum pump. This step is repeated with all the nonwoven layers that are placed cross-oriented. Finally, the mold is filled with a layer of cement paste with the same content of the first cement layer. After finishing this process, the composite is compacted for 10 min with a pressure of 3.5 MPa to obtain a plate with a thickness of about 8–12 mm. An image of the mold (with internal dimensions of 300 × 300 × 30 mm^3^) and the pressing process is presented in Figure 8.

The demolded plates were then cured for 28 days at room temperature (20 °C), in a humidity chamber (approximately 95% of relative humidity). From the plates, six specimens of (8~12) × 45 × 300 mm^3^ were machined using a diamond saw. 

To analyze the infiltration of the cement through the nonwoven structures, backscattered electron (BSE) images at 15 kV and 20 kV were obtained from the cut, polished, and covered with an epoxy resin surfaces. This technique allows the identification of the composite phases. A JEOL JSM-6300 microscope (Jeol Ltd., Tokyo, Japan) was used.

To determine the flexural behavior, four-point bending tests were carried out following the RILEM TFR1 [33] and TFR4 [34] standard test, using an Incotecnic press at a cross-head speed of 2 mm/min. The major span (L) was 270 mm, and the displacement measurements were carried out using two LVDTs of 0.01 mm of resolution, and an error of 0.15%. 

Direct tensile tests were performed at a cross-head speed of 0.1 mm/min. The span between grips was 200 mm. 

The load cells used for the bending test and the tensile tests were respectively 3 kN and 20 kN. The following parameters were obtained from the curves of these tests: limit of proportionality (LOP), defined as the stress value at the upper point before appears the first crack; modulus of rupture (MOR), which corresponds to the maximum stress supported by the material, reached either by breaking or by obtaining a displacement of more than 10% of the separation between the span supports; E_zone_ or modulus of stiffness, calculated as the relationship between the stress and strain between two points on the stress-strain curve. This value has been calculated for the initial phase of the curve before reaching the LOP or zone A (E_A_), for the first post-failure zone or zone B (E_B_) and for the crack propagation zone or zone C (E_C_). Finally, the specific energy absorbed by the material during the test or toughness was calculated as the area under the curve force versus displacement from the starting to the value of the ordinate corresponding to a reduction higher than 40% of the MOR or a span higher than 10%, divided by the areal cross section of the specimen.

## 4. Conclusions 

The following conclusions can be drawn from this research:

- The use of nonwoven flax fabrics as reinforcement in cement-based composites leads to cement materials with very high ductility.
- Nonwoven structures with low thickness and high entanglement, in the form of multilayer reinforcement, allow a higher infiltration of the cement paste through the nonwoven promoting higher fiber-matrix adherence.
- Increasing the number of nonwoven layers from three to four leads to an increase of the flexural MOR and toughness values of 19.7% and 26.9% respectively. This increase is of 49.1% and 46.7% of the flexural MOR and toughness values respectively for the composites prepared with the water treated nonwovens.
- The composites reinforced with the nonwovens with high entanglement (HNP-NW) presented higher values of flexural LOP (226%), MOE (7%), MOR (7%), and toughness (28%) than the ones prepared with the nonwovens with lower entanglement (MNP-NW). A similar trend was found for the LOP, MOE, MOR, and toughness values determined under tensile configuration with increases of 90%, 8%, 11%, and 20%, respectively.

## Figures and Tables

**Table 1:** Sample reference, composition, and values of flexural strength and toughness of the composites prepared to analyze the effect of number of layers and the water treatment of the nonwoven fabrics.

Sample Reference	Number of Layers	Nonwoven Treatment	Nonwoven: Cement Ratio (wt %)	Final w/c Ratio (after Mold Compression)	Flexural Strength (MPa)	Toughness (kJ/m^2^)
C-3LNW	3 layers	Untreated	6.7	0.50	13.9 ± 2.22	4.9 ± 0.24
C-4LNW	4 layers	Untreated	9.1	0.48	17.3 ± 3.34	6.7 ± 0.34
C-4L-NW_T	4 layers	Water treatment	9.1	0.43	27.3 ± 0.59	9.2 ± 0.17

**Table 2:** Breaking force and deformation obtained from the tensile tests and weight and thickness of the high needle-punched (HNP-NW) and medium needle-punched (MNP-NW) nonwoven fabrics prepared as reinforcement for the cement composites.

Reference	Test Direction	Maximum Tensile Force (N)	Deformation (%)	Weight (g/m^2^)	Thickness (mm)
HNP-NW	MD ^1^	40.7 ± 17.1	43.3 ± 6.8	-	-
	CD ^2^	41.2 ± 15.2	52.7 ± 6.7	-	-
	Average	40.9 ± 16.1	48.0 ± 6.7	276	1.2 ± 0.1
MNP-NW	MD	12.8 ± 1.9	31.3 ± 5.9	-	-
	CD	22.9 ± 9.3	48.4 ± 8.4	-	-
	Average	17.8 ± 5.6	39.8 ± 7.1	213	1.6 ± 0.1

^1^ MD: machine direction; ^2^ CD: cross direction.

**Table 3:** Average values and standard deviation of LOP, MOR, E modulus, and toughness, obtained from bending tests and increase of these values for the composites prepared with the HNP-NW fabric with respect to the ones prepared with the MNP-NW fabrics.

Parameter	MNP-NW Composite	HNP-NW Composite	Increase for HNP-NW vs. MNP-NW Composite
LOP (MPa)	5.54 ± 0.99	18.05 ± 4.59	226%
MOR (MPa)	31.08±1.61	33.41 ± 4.35	7%
Toughness (kJ/m^2^)	13.99 ± 1.62	16.68 ± 1.59	28%
E_A_ (GPa) (zone A)	11.09 ± 3.66	15.71 ± 0.65	42%
E_B_ (GPa) (zone B)	0.31 ± 0.26	0.517 ± 0.06	69%
E_C_ (GPa) (zone C)	0.33 ± 0.21	0.338 ± 0.18	2%

**Table 4:** Average values and standard deviation of LOP, MOR, E modulus, and toughness, obtained from tensile tests and increase of these values for the composites prepared with the HNP-NW fabric with respect to the ones prepared with the MNP-NW fabrics.

Parameter	MNP-NW Composite	HNP-NW Composite	Increase of Property HNP-NW vs. MNP-NW Composite
LOP (MPa)	2.67 ± 1.43	5.06 ± 2.91	90%
MOR (MPa)	7.18 ± 0.94	7.75 ± 0.34	8%
Toughness (kJ/m^2^)	35.49 ± 3.27	39.34 ± 3.51	11%
E_A_ (GPa) (zone A)	15.08 ± 0.98	18.17 ± 3.47	20%
E_B_ (GPa) (zone B)	0.14 ± 0.11	0.16 ± 0.15	8%
E_C_ (GPa) (zone C)	0.14 ± 0.16	0.15 ± 0.12	8%

## Supplementary Materials

The following are available online at www.mdpi.com/1996-1944/10/2/215/s1. Video S1: Flexural behavior.
